# Evaluating Potential Therapies in a Mouse Model of Focal Retinal Degeneration with Age-related Macular Degeneration (AMD)-Like Lesions

**DOI:** 10.4172/2155-9570.1000296

**Published:** 2013-09-23

**Authors:** Nicholas Popp, Xi K. Chu, Defen Shen, Jingsheng Tuo, Chi-Chao Chan

**Affiliations:** Immunopathology Section, Laboratory of Immunology, National Eye Institute, National Institutes of Health, 10 Center Dr., 10/10N103, NIH/NEI, Bethesda, MD, 20892-1857, USA

**Keywords:** Age-related macular degeneration (AMD), Mouse models, *Ccl2*, *Cx3cr1*, *rd8*, Therapy

## Abstract

Although the mouse has no macula leutea, its neuroretina and retinal pigment epithelium (RPE) can develop lesions mimicking certain features of age-related macular degeneration (AMD). Differences between the *Ccl2* and *Cx3cr1* double deficient mouse on *Crb1^rd8^*(*rd8*) background (DKO^*rd8*^) and the *Crb1^rd8^* mouse in photoreceptor and RPE pathology, as well as ocularA2E contents and immune responses, show that DKO^*rd8*^ recapitulates some human AMD-like features in addition to *rd8* retinal dystrophy/degeneration. Different therapeutic interventions have been demonstrated to be effective on the AMD-like features of DKO^*rd8*^ mice. The use of the DKO^*rd8*^ model and C57BL/6N (wild type, WT) mice as group controls (4 groups) to test treatments such as high omega-3 polyunsaturated fatty acid (n-3) diet has, for example, shown the beneficial effect of n-3 on AMD-like lesions by anti-inflammatory action of docosahexaenoic acid (DHA) and eicosapentaenoic acid (EPA). The use of self-control in the DKO^*rd8*^ mouse by treating one eye and using the contralateral eye as the control for the same mouse allows for appropriate interventional experiments and evaluates various novel therapeutic agents. Three examples will be briefly presented and discussed: (1) tumor necrosis factor-inducible gene 6 recombinant protein (TSG-6) arrests the AMD-like lesions via modulation of ocular immunological gene expression, e.g., Il-17a; (2) adeno-associated virus encoding sIL-17R (AAV2.sIL17R) stabilizes the AMD-like lesions; and (3) pigment epithelium-derived factor (PEDF) ameliorates the AMD-lesions by its anti-inflammatory, anti-apoptotic and neuroprotective roles. Therefore, the DKO^*rd8*^ mouse model can be useful and appropriate for therapeutic compound screening in the management of human AMD.

## Introduction

Age-related macular degeneration (AMD) has become the leading cause of irreversible central vision loss in the elderly, affecting approximately 7.2 million people in United States over the age of 40 (6.5%) [1]. AMD is also the third leading cause of blindness globally, after cataracts and glaucoma [2]. Most patients with AMD gradually start to lose central vision after age 60, and though many individuals are affected by the disease, treatment options are few [3]. Currently, new treatment development is limited by a lack of understanding of AMD pathogenesis and is complicated by its late onset, complex genetics, and the influence of environmental risk factors such as age, smoking, and diet [3–5].

To better understand the complex etiology of AMD, genetically engineered mouse models have been developed to study the molecular effects of AMD on the retina. Although the mouse has no macula, its eyes can develop many characteristics of AMD, including focal atrophy of the photoreceptors, retinal pigment epithelium (RPE) degeneration, lipofuscin accumulation, and increased ocular A2E levels [6–8]. AMD is a complicated disease involving multiple etiologies, including oxidative damage, immune dysregulation, and lipid metabolism dysfunction, and many mouse models have been developed to study these pathways. While this review will only focus on mouse models of immune dysregulation including *Ccl2^−/−^, Cx3cr1^−/−^*, and DKO^*rd8*^, many other models exist and have been excellently reviewed elsewhere [7,9]. Immune dysregulation animal models are useful for their presentation of AMD-like characteristics and for testing therapeutic compounds.

## Development of DKO^*rd8*^ as a Murine Model of AMD

Evidence of immune involvement in AMD [10] has led to the development of a mouse model knockout of monocyte chemoattractant protein-1 (MCP-1/CCL2) or its cognate chemokine receptor (CCR2) [11]. This ligand-receptor interaction allows macrophages to adhere to the walls of blood vessels, thus controlling their recruitment to tissue [7,12–14]. It is believed that CCL2 may function as a neuroprotective factor against apoptosis [8,14]. Senescent *Ccl2* or *Ccr2* deficient mice produce several features of human AMD, including photoreceptor atrophy, lipofuscin accumulation, and drusen formation. In *Ccl2* deficient mice, RPE degeneration, thickening of the Bruch’s membrane, and complement activation are seen by 9 months of age [11].

Genetic epidemiology studies revealed *CX3CR1* loss of function variants to be associated with AMD and functional study, furthermore, showed lower CX3CR1 expression in the macula compared to peripheral retina of AMD patients [15]. CX3CR1 is a chemokine receptor involved in recruiting inflammatory cells to the retina to eliminate macular deposits; loss of function of this chemokine results in photoreceptor damage arising from the inability to clear the deposits from the macula [15–17]. In addition, CX3CR1 expressing cells (mostly microglia) are found to accumulate in the subretinal space of the macula, leading to an inflammatory environment, which results in pathological damage. The *Cx3cr1* deficient mice also develop AMD-like features in the retina [16].

Based on these compelling factors, a *Ccl2^−/−^/Cx3cr1^−/−^* double knockout mouse model (DKO) was generated to determine whether deficiencies in both genes might together produce more characteristic and more reproducible features of AMD in a mouse model [8,18]. Significantly, the DKO model has earlier onset and higher penetrance than the two single knockout models of *Ccl2* and *Cx3cr1*. DKO shows multiple small retinal lesions by 4–6 weeks of age, comparable to the focal retinal lesions in human AMD, in addition to RPE degeneration, A2E elevation, and aberrant complement deposition [8,18]. These features are highly reproducible, making the DKO model more suitable for AMD research [6].

Recently, it was found that all C57BL/6N mice, including the *Ccl2^−/−^, Cx3cr1^−/−^*, and DKO mouse models generated from this mouse strain, contain a homozygous frame shift mutation in the *Crumbs homolog 1* gene (*Crb1^rd8^*) and that this mutation leads to retinal degeneration [19,20]. Crb1 is a molecular scaffolding protein and is shown to be highly associated with disease phenotype [20,21]. However, detailed study has shown that the DKO model on this *Crb1^rd8^* background (DKO^*rd8*^) presents certain disease pathology that is characteristic of AMD and differs notably from *Crb1^rd8^* alone [6]. At two months of age, *Crb1^rd8^* mice exhibit retinal folds and pseudorosettes, photoreceptor inner and outer segment shortening, and photoreceptor dystrophy [6,21]. In contrast, age-matched DKO^*rd8*^, typically show no retinal folds and pseudorosettes but exhibit RPE cell alteration, A2E elevation, and increased macrophage infiltration and complement activation, all of which are primary markers of AMD that are not readily seen in *Crb1^rd8^* [6]. Because this model recapitulates many of the key morphological and immunological characteristics of AMD, DKO^*rd8*^ can be used to study AMD pathogenesis as well as to test new therapeutic compounds. Furthermore, *Ccl2^−/−^/Cx3cr1^−/−^* mice that were generated from C57BL/6J (without *Crb1^rd8^*) can also develop localized retinal atrophy, similar to human geographic atrophy AMD [22].

**Figure 1:**
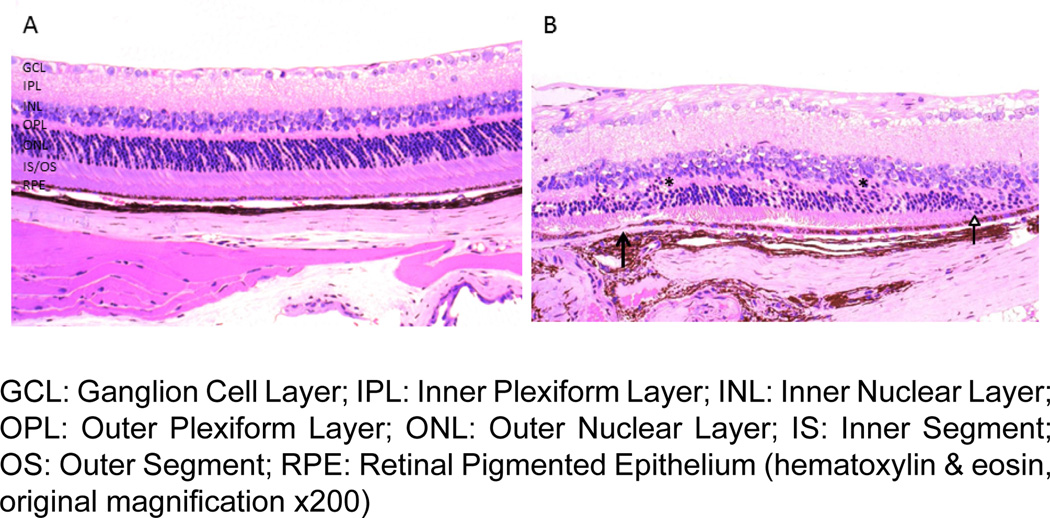
Photomicrograph of DKO^*rd8*^ and DKO retinas. (A) DKO without *Crb1^rd8^* background does not show photoreceptor shortening in the IS/OS. The RPE is unremarkable. (B) DKO^*rd8*^ shows RPE degeneration (black arrow), shortening of the IS/OS and photoreceptor degeneration (white arrow), and *rd8*-associated lesions (asterisks).

**Figure 2:**
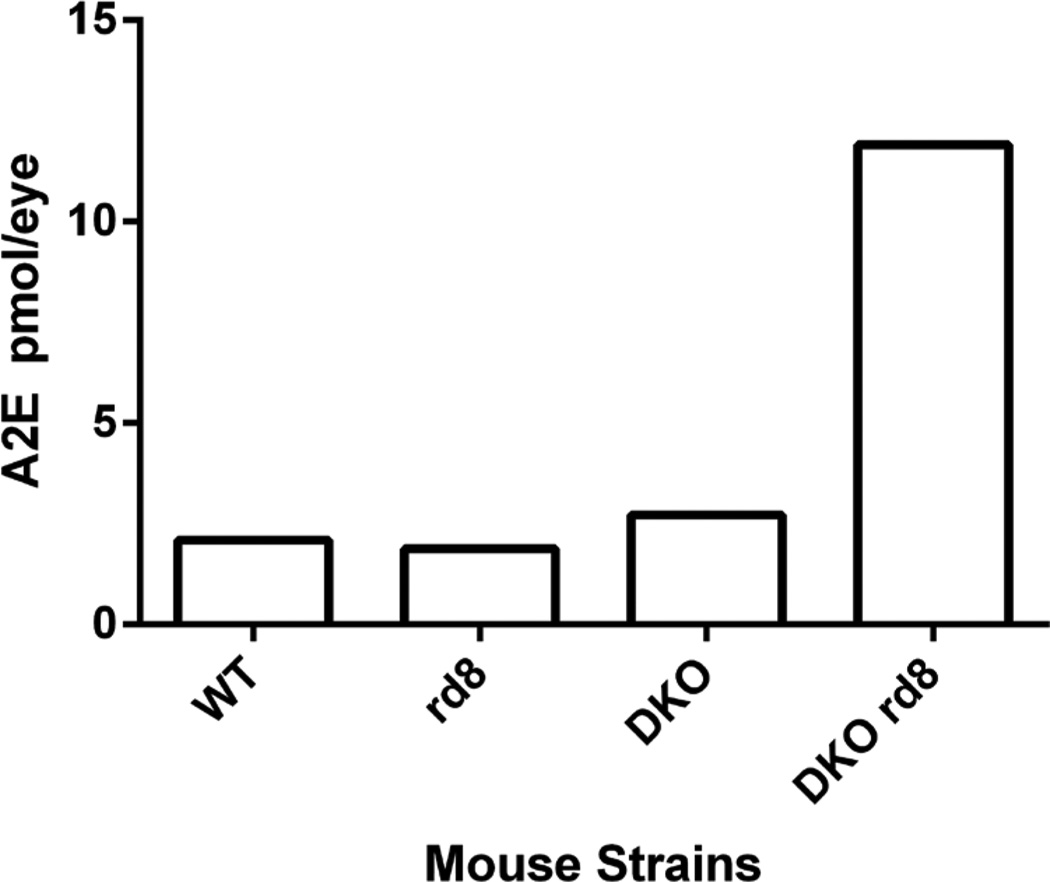
Quantification of A2E in WT, *Crb1^rd8^*, DKO, DKO^*rd8*^ strains at 3 months. DKO^*rd8*^ exhibits a five-fold increase in A2E per eye compared to the other three strains.

Some authors have since argued that DKO^*rd8*^ may not be a model for AMD, suggesting the *Crb1^rd8^* mutation, instead of mutations in *Ccl2 and Cx3cr1*, is the source of the AMD-like pathology found in the DKO^*rd8*^ model [23–25]. However, retinal histology from DKO without *Crb1^rd8^* background does not show the typical photoreceptor degeneration in the inner and outer segments (IS/OS) that are seen in DKO^*rd8*^ (Figure 1A). DKO^rd8^ also shows *rd8*-associated lesions and RPE degeneration (Figure 1B). In addition to these structural changes, A2E levels in the eyes of DKO^*rd8*^ mice are five-fold higher than in the eyes of wild type (WT), *Crb1^rd8^*, or DKO without *Crb1^rd8^* background mice (Figure 2). These and other differences between DKO, DKO^*rd8*^, C57BL/6N (with *Crb1^rd8^* background), and *Crb1^rd8^* mouse models are described in Table 1. The differences in A2E levels, spontaneous retinal degeneration, and RPE dysfunction between these three models all highlight the importance of the genetic background of the mouse. C57BL/6N genetic background is important to allow for full expression of the *rd8* phenotype [20]. Our data, in conjunction with other reports, suggest that the C57BL/6N genetic background is also important in the phenotypes seen in DKO^*rd8*^ [22,23,25].

## Evaluating Therapeutic Interventions using DKO^*rd8*^

**Figure 3:**
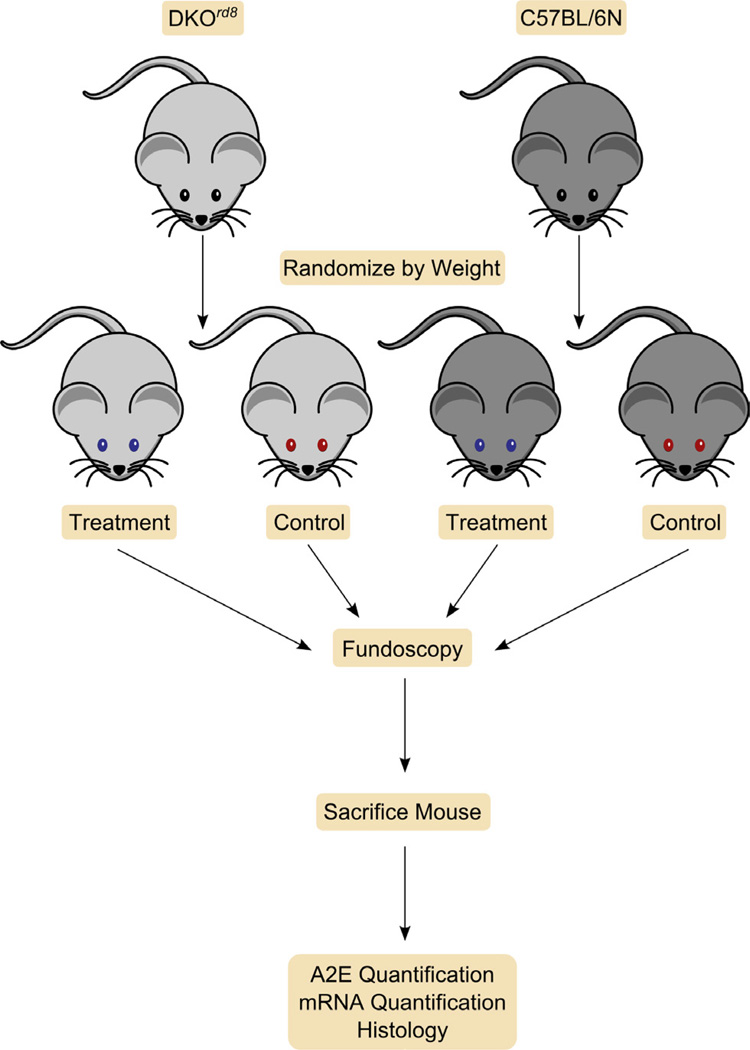
Group Controls In group control experiments, DKO^*rd8*^ and C57BL/6 (WT) mice are ramdomized by weight and separated into treatment or control groups. The 4 groups of mice are followed by fundoscopy for certain duration (usually 2–3 months), then sacrificed, and the retinal samples are analyzed for AMD biomarkers including A2E levels, histopathology, and quantification of various mRNA transcripts. (blue eyes, received treatment; red eyes, controls that received placebo or left untreated)

To date, DKO^*rd8*^ has been used in both group control experiments and self-control experiments (Figure 3). One example of a group control design is the use of DKO^*rd8*^ in assessing the effect elevated omega-3 (n-3) fatty acids on AMD progression.

Epidemiological studies have indicated that increased intake of omega-3 (n-3) fatty acids may have a protective role in countering development of advanced AMD [4,26–28]. In the Age-Related Eye Disease Study (AREDS), a 12 year follow-up study of over 4,000 AMD patients, those who reported the highest intake of n-3 long chain polyunsaturated fatty acids (LCPUFAs) were 30% less likely to develop advanced AMD than were those who reported the lowest levels of intake of n-3 fatty acids [29]. The AREDS prompted a large, randomized clinical trial (AREDS2) where patients were given varying formulations of LCPUFAs: lutein and zeaxanthin, docosahexanenoic acid (DHA) and eicosapentaenoic acid (EPA), or both formulations simultaneously [30]. In addition, all patients were also prescribed the AREDS formula of high anti-oxidative agents including vitamins C and E with zinc and copper. Through a secondary randomization process, some patients were given beta-carotene (as in the original formulation) whereas others were not. Patients given the AREDS2 formulas with lutein and zeaxanthin with or without DHA and EPA showed a decrease in the progression to advanced AMD. Further, patients given lutein and zeaxanthin without beta-carotene showed a significant drop in disease progression. Interestingly, this beneficial effect was most pronounced for the quintile given the lowest levels of lutein and zeaxanthin [30].

An early mouse study of the therapeutic potential of n-3 fatty acids on AMD focused on DHA and EPA, which are typically found in high concentration in the retina [31]. DKO^*rd8*^ mice were used as a diseased population, subdivided into DHA/EPA treatment (high n-3 fatty acids) and placebo groups (low n-3 fatty acids). C57BL/6N mice were not included in the feeding experiment, as they do not show AMD-like lesions at any age [8,18,31]. At 27 weeks, 90% of high n-3 diet DKO^*rd8*^ showed lesion regression. At the same age, only 16% of low n-3 diet DKO^*rd8*^ showed the same lesion regression. RPE degeneration and retinal A2E was also higher in the low n-3 fatty acid-fed DKO^*rd8*^ mice [31]. This evidence suggests that, in the DKO^*rd8*^ murine model, inflammatory response is reduced by the introduction of high levels of n-3 fatty acids to the diet and may serve as a protective factor for the progression of AMD-like retinal lesions.

In a separate experiment, DKO^*rd8*^ and C57BL/6N with *Crb1^rd8^* background (WT) mice were given the AREDS2 dosages of lutein, zeaxanthin, DHA, and EPA (treatment) or fed an isocaloric diet (control). C57BL/6N mice for both treatment and control groups showed no AMD-like lesions. In contrast, treated DKO^*rd8*^ mice showed significantly higher AMD-like lesion regression compared to DKO^*rd8*^ controls which were more likely to progress to more severe AMD symptoms based on fundoscopies [32]. Histopathology confirmed these findings, where treatment of DKO^*rd8*^ mice prevented degeneration of retinal architecture and photoreceptor loss. Additionally, treated DKO^*rd8*^ mice showed similar levels of A2E biomarker as WT mice, which was significantly lower than the control DKO^*rd8*^ mice [32].

These results from the two murine model studies suggest that n-3 fatty acids play a protective role in the pathogenesis of AMD. However, the AREDS2 human study suggests that lutein and zeaxanthin, not n-3 fatty acids, are the primary beneficial dietary supplements for the alleviation of AMD. These results are interesting and require further study to determine the underlying cause of these differences. However, these findings do serve as a reminder that results from treatment research on a murine model may not directly translate to human patients.

**Figure 4:**
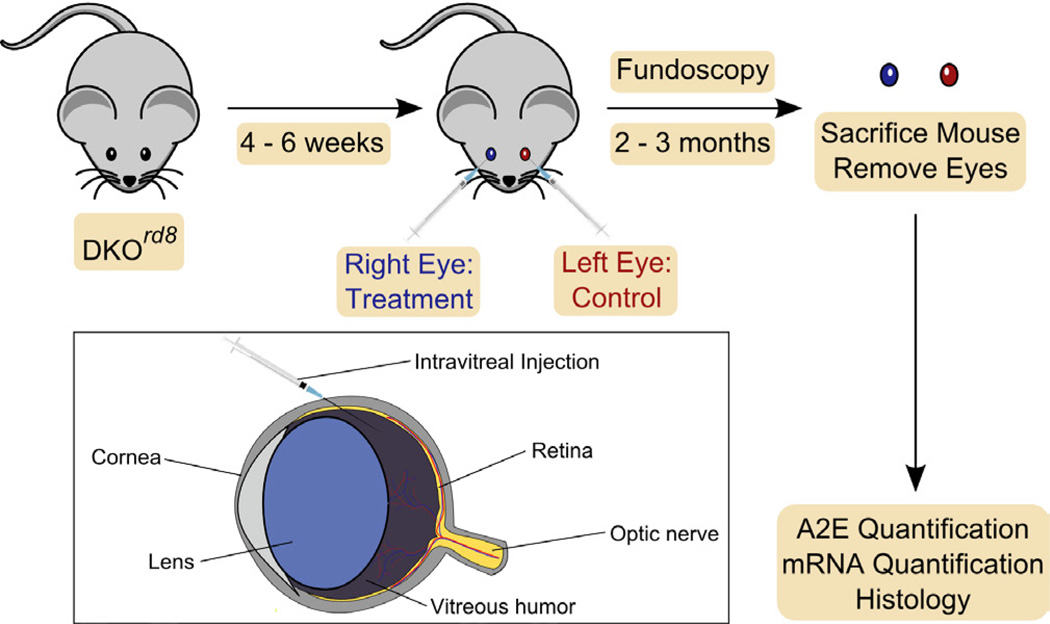
Self Controls In self control experiments, DKO^*rd8*^ mice are treated intraocularly with a therapeutic agent in one eye (in blue) while the other (contralateral) eye (in red) is treated with control agent (placebo) or left untreated. Mice are followed by fundoscopy for certain duration (usually 2–3 months), then sacrificed, and the retinal samples are analyzed for a variety of AMD biomarkers and histology. Inset: Magnified schematic of intravitreal injection. Treatment or placebo are injected into the vitreous humor of the mouse eye where it can diffuse to the retina.

While DKO^*rd8*^ does have some limitations as a model of AMD given the absence of macula in mice and an incomplete understanding of *Crb1^rd8^* and *Ccl2^−/−^/Cx3cr1^−/−^*, using the same mouse as a self-control is ideal for studying the effects of novel ocular treatments [6]. Following this design, a single mouse serves as both the experimental and control situations by applying treatment directly to one eye and applying a control to the contralateral eye (Figure 4). Because each mouse has a control and treatment eye, variations between mice (e.g. genetic background) that can confound group experiments are removed from this design. The results are then averaged over a cohort of mice to determine a final effect of treatment and control. The following three experiments have been conducted successfully in this manner using the DKO^*rd8*^ murine model.

Tumor necrosis factor-inducible gene 6 protein (TSG-6) is an anti-inflammatory protein that has been used in other mouse models to reduce inflammation in the heart and cornea [32–38]. Since increased macrophage infiltration and complement activation have been linked to AMD disease progression, TSG-6 was administrated intravitreally to determine if it could have beneficial effect on retinal lesions in DKO^*rd8*^ [39]. Compared to the phosphate-buffered saline control injected into the left eyes of the mice, the right eyes showed lesion arrest whereas the untreated left eyes showed worsening lesions overtime. Microarray data from the retina showed decreased expression of the inflammatory Tnf-α and Il-17a, suggesting an inhibition of inflammatory damage associated with AMD pathology. Interestingly, A2E levels were unchanged, suggesting that the value of TSG-6 treatment comes mainly from anti-inflammatory effects rather than inhibition of oxidative stress [39]. These findings suggest that AMD patients may benefit from TSG6 supplements.

The TSG-6 study indicated the possible involvement of IL-17A in inflammatory damage of AMD, a hypothesis that was further supported by evidence of elevated IL-17 in the sera of AMD patients as compared to controls [40]. Furthermore, increased levels of *IL-17A* mRNA and protein were found in the macular lesions of patients with AMD compared to controls [41]. These data together suggest IL-17A could play a key role in the damage caused by AMD and that localized knockdown of this protein may lead to amelioration of the disease.

At 6 weeks of age, adeno-associated virus containing a soluble IL-17 receptor (AAV2.sIL17R) was injected into the right eyes of DKO^*rd8*^ mice and an empty vector (AAV2.EV) into the left eyes. Clinically, AAV2.sIL17R retinas showed improvement, exhibiting lower levels of A2E, a reduced number of lesions, and less retinal degeneration compared to AAV2.EV eyes [42]. Importantly, knockdown of Il-17a did not prevent or improve *rd8* retinal dystrophy, suggesting IL-17A to be a specific target for AMD-like retinal degenerative disease and highlighting the phenotype of DKO^*rd8*^ to be that of retinal degeneration rather than retinal dystrophy alone [42].

Pigment Epithelium-Derived Factor (PEDF) is a pleiotropic glycoprotein found in many tissues, including the retina. Although PEDF effects throughout the body can range from anti-angiogenic to pro-apoptotic, it functions as a neurotrophic and anti-inflammatory agent in the retina [43–48]. PEDF has been shown to protect the RPE and photoreceptors against cell death from various pathological insults. As such, it was hypothesized that injection of PEDF may decrease lesion progression in DKO^*rd8*^ mice. Six-week old mice were injected intravitreously with recombinant human PEDF in one eye while the other was left untreated for control [49]. Deep focal retinal lesions progressed more slowly or attenuated in PEDF-treated eyes compared to control. In addition, there were fewer photoreceptor lesions, significantly lower A2E levels, and lower expression of apoptotic and pro-inflammatory transcripts (*Tnf-α, Il-17a,Il1*β mRNAs) in the PEDF-treated eyes [49]. These results underscore the role of inflammation in AMD and suggest using PEDF as a new potential treatment therapy for AMD.

## Conclusions

Despite some limitations to studying macular degeneration in murine models, a *Ccl2^−/−^/Cx3cr1^−/−^* double knockout on *Crb1^rd8^* background (DKO^*rd8*^) reliably reproduces many of the key features of human AMD: focal photoreceptor and RPE pathology, increased levels of the lipofuscin biomarker A2E, and aberrant immune activation including macrophage infiltration and complement deposition [7,8,10]. While the *rd8* mutation in the *Crb1* gene is needed for the DKO^*rd8*^ phenotype, this mutation alone may not be sufficient to recapitulate all manifestations of AMD [6,8,18,20]. Instead, *Crb1^rd8^* mainly leads to retinal dystrophy in early ages. The lesions in DKO^*rd8*^ show improvement with intervention studies utilizing both group control (n-3 fatty acid and AREDS2 diets) [31] (Figure 3) or self-control (TSG-6, AAV2.sIL17R, and PEDF) experimental designs [39,42,49] (Figure 4). However, it is possible that clinical trial data on AMD patients may not yield identical results as on the DKO^*rd8*^ murine model, as was the case with the AREDS2 study [30,32]. Taken together, these findings indicate that while caution must always be taken in translating findings in mouse models to findings in humans, the AMD-like phenotype of DKO^*rd8*^ makes the model a highly useful tool for screening potential therapies for AMD.

## Figures and Tables

**Table 1:** Retinal degeneration and immune activation in DKO^*rd8*^, DKO, *Crb1^rd8^*, and C57BL/6N (with *Crb1^rd8^* background) strains.

Mouse Strain	DKO^*rd8*^ *Ccl2^−/−^/Cx3cr1^−/−^/Crb1^rd8^*	DKO *Ccl2^−/−^/Cx3cr1^−/−^Crb1^+/+^*	*Crb1^rd8^*	C57BL/6N (*Crb1^rd8^*background)
***Rd8* Retinal Degeneration**	+	−	+	+ (rare)
**AMD-like Retinal Degeneration**				
**Onset**	2–6 weeks	12 months	2–3 months	2–3 months
**Spontaneous**	+	−	+	−
**Photoreceptor Shortening**	++	+ (induced)	+ (late)	−
**RPE Degeneration**	+	+ (induced)	−	−
**Elevated A2E**	+	−	−	−
**Macrophage Infiltration**	++	+ (late)	−	−
**Complement Deposition**	++	++ (late)	−	−
**References**	[6,8]	[22]	[6]	[6,20]
